# Isoflurane and Surgical Stress Disrupt Fatty Acid and Carbon Metabolism, Leading to Cardiomyopathy in Aged Mice

**DOI:** 10.3390/cells15030237

**Published:** 2026-01-26

**Authors:** Wendy W. Yang, Anna W. Chen, Hangnoh Lee, Hui Li, Jin-Gu Lee, Yun Li, Wei-Bin Shen

**Affiliations:** 1 Department of Anesthesiology, University of Maryland School of Medicine, Baltimore, MD 21201, USA; 2 Department of Obstetrics, Gynecology & Reproductive Sciences, University of Maryland School of Medicine, Baltimore, MD 21201, USA; 3 Department of Medicine, University of Maryland School of Medicine, Baltimore, MD 21201, USA

**Keywords:** anesthesia/surgery, aging, bulk RNA sequencing, heart, longitudinal RNAseq profiling

## Abstract

**Highlights:**

**What are the main findings?**

- Acute Iso/Op exposure triggers age-dependent disruption of cardiac metabolic, calcium-handling, and structural gene programs.
- Isoflurane drives sustained cardiac transcriptomic reprogramming that persists five weeks after exposure in 20-month-old mice.

**What are the implications of the main findings?**

- Age should be considered a critical biological modifier of cardiac responses to perioperative anesthetic and surgical stress.
- Isoflurane exposure may contribute to long-term cardiac molecular vulnerability beyond the immediate postoperative period.

**Abstract:**

Aging alters cardiac resilience to anesthetic and surgical stress, yet the molecular basis for these effects remain poorly understood. To define age-dependent transcriptional responses, we profiled cardiac gene expression across young adult (3 m), late middle-aged (17 m), and old mice (27 m) following 2 h isoflurane and operative (Iso/Op) exposure. Across all age groups, 24 h after cessation, Iso/Op induced distinct transcriptional signatures relative to the sham, with conserved perturbations in oxidative stress responses, Ca^2+^ handling, hypertrophy-associated signaling, and energy metabolism. In 3 m hearts, transcriptional alterations were characterized by dysregulation of small-molecule catabolism, fatty acid metabolism, endoplasmic reticulum processing, and cytoskeletal organization. In 17 m hearts, lipid metabolic disruption was amplified and accompanied by suppression of muscle system and calcium signaling pathways. In 27 m hearts, Iso/Op robustly activated PPAR and AMPK signaling and fatty acid catabolic programs while downregulating pathways governing contractility, actin organization, and morphogenesis, consistent with age-associated maladaptive metabolic reprogramming. To assess persistence, we analyzed a longitudinal cohort of 20 m mice five weeks after exposure and observed sustained transcriptomic remodeling driven predominantly by isoflurane, including mitochondrial dysfunction and altered expression of genes linked to diabetic cardiomyopathy, extracellular matrix integrity, and neurodegeneration-associated pathways. Together, these data suggest that isoflurane-based perioperative stress can produce age-amplified and durable metabolic and structural cardiac remodeling, implicating impaired lipid utilization and mitochondrial homeostasis as potential mechanisms of long-term cardiovascular vulnerability.

## 1. Introduction

General anesthesia (GA) is essential for surgery and diagnostic procedures, yet it can exert systemic effects on the cardiovascular system. Evidence indicates that GA and surgery can trigger systemic metabolic and inflammatory responses that can persist beyond the perioperative period. Perioperative neurological deficits (PND) increase markedly with aging [1,2,3,4,5,6,7]; however, the cardiovascular consequences of anesthesia and surgical stress, particularly in aging patients undergoing noncardiac procedures, remain poorly understood [8,9]. GA and surgery activate systemic inflammation, oxidative stress, and neuroendocrine pathways [10,11,12,13], which may secondarily but meaningfully impact cardiac tissue. Isoflurane (Iso), a widely used volatile anesthetic, is generally well tolerated in young hearts and may confer ischemic preconditioning [14,15], however, in aged hearts, it has been linked to mitochondrial dysfunction, altered substrate utilization, and increased oxidative stress, potentially affecting transcriptomic profiles during recovery [16,17]. Consistent with these findings, prior studies report Iso-induced cardiac depression and oxidative stress [18], as well as dose-dependent alterations in cardiac hemodynamics with occupational exposure [19]. Despite these observations, the transcriptional impact of short-term isoflurane exposure combined with surgical operation (Iso/Op) on the heart has not been systematically examined. Given that age-related mitochondrial dysfunction and oxidative stress drive persistent transcriptional remodeling, we hypothesized that even brief Iso/Op exposure could induce lasting cardiac gene expression changes in aged hearts.

Aging is a major risk factor for adverse cardiovascular outcomes in the perioperative setting. Older adults exhibit increased susceptibility to myocardial injury, arrhythmias, and delayed recovery following anesthesia and surgical stress [20,21]. This vulnerability reflects progressive declines in cardiac metabolic flexibility, mitochondrial efficiency, and redox homeostasis that limit the heart’s ability to adapt to acute energetic and oxidative stressors. Age-related remodeling of cardiomyocyte bioenergetics includes impaired fatty acid oxidation, altered NAD^+^ metabolism, and increased reliance on glycolytic substrates, collectively limiting ATP availability under stress [22,23,24]. Although these physiological vulnerabilities are well established, the molecular and transcriptional mechanisms by which aging alters cardiac responses to anesthesia and surgical stress remain poorly defined. Understanding these age-dependent pathways is crucial for identifying therapeutic targets to reduce postoperative cardiac dysfunction.

Our previous transcriptomic analyses demonstrated that Iso/Op exposure in aged mice induces neural and behavioral impairments, causing persistent olfactory deficits, motor frailty, and delayed cognitive decline [25], suggesting that perioperative stress can induce lasting CNS changes. Whether comparable age-dependent transcriptional remodeling occurs in peripheral, metabolically demanding organs such as the heart remains unknown. Given emerging evidence of metabolic coupling between the heart and other organ systems [26,27], understanding how GA and surgery affect cardiac bioenergetics is crucial for uncovering mechanisms of perioperative vulnerability in aging. Notably, experimental models show that cardiac dysfunction can alter hippocampal gene expression and impair memory, underscoring the heart’s influence on cognition [28].

Although age affects anesthetic sensitivity, how GA and surgery reprogram cardiac transcription across the lifespan remains unclear. Recent transcriptomic advances allow high-resolution mapping of age-dependent molecular stress responses. To model systemic perioperative stress while minimizing direct neurovascular injury, we employed a standardized laparotomy paradigm. By design, procedures involving the direct manipulation of central or peripheral nerves, as well as major arteries or veins were excluded a priori. This approach provides a well-characterized systemic stress model while minimizing direct neural and vascular manipulation. Here, we investigated how aging alters the magnitude, direction, and persistence of cardiac transcriptional responses to short-term Iso/Op. We hypothesized that aging alters the temporal and pathway-specific transcriptional responses to Iso/Op, leading to distinct molecular signatures of metabolic and structural remodeling. To isolate age-dependent effects on cardiac transcriptional remodeling, the present study intentionally employed a single, widely used anesthetic–surgical paradigm (isoflurane combined with laparotomy), rather than comparing multiple anesthetic agents. By integrating age-stratified transcriptomic analyses, we define dynamic, age-dependent cardiac transcriptional programs that may underlie heightened perioperative vulnerability in the aged heart. We profiled both acute (24 h) and long-term (5 w) transcriptional responses to assess how aging affects cardiac resilience to perioperative stress. Our results reveal that Iso/Op rapidly induces age-dependent dysregulation of fatty acid metabolism, cytoskeletal integrity, and mitochondrial pathways, with lasting changes in genes linked to diabetic cardiomyopathy and neurodegeneration. Together, these findings identify perioperative isoflurane-based stress as a driver of metabolic and structural remodeling in the aging heart, shedding light on perioperative cardiac vulnerability.

## 2. Materials and Methods

### 2.1. Mouse Isoflurane Exposure and Operation (Iso/Op)

All animal experiments were conducted in accordance with protocols approved by the Institutional Animal Care and Use Committee (IACUC) at the University of Maryland School of Medicine (AUP-00000092). Young adult (10–12 weeks, 2.5–3.0-month-old), middle-aged (17-month-old), geriatric (20–21-month-old), and old (27-month-old) male C57BL/6 mice were sourced from Charles River Laboratories through the National Institute on Aging (NIA). Mice were housed under a 12 h light/dark cycle with ad libitum access to food and water. Within each age group, mice of the same age group were randomly assigned to either the Sham or Iso/Op condition. This design yielded six experimental groups: 3-month Sham (*n* = 5 mice) and Iso/Op (*n* = 5 mice), 17-month Sham (*n* = 3 mice) and Iso/Op (*n* = 3 mice), and 27-month Sham (*n* = 5 mice) and Iso/Op (*n* = 5 mice). General anesthesia was induced and maintained with 2% isoflurane delivered in 100% oxygen via a precision vaporizer. Anesthetic depth was monitored by respiratory rate and depth, as well as palpebral and pedal responses. Core body temperature was maintained at physiological levels using a controlled heating pad throughout the procedure. After induction, mice were positioned on a surgical station with a nose cone for continuous isoflurane delivery. A midline laparotomy was performed through a 1.5 cm incision extending from the xiphoid process to approximately 0.5 cm above the pubic symphysis, sequentially cutting through the skin, abdominal musculature, and peritoneum. The incision was infiltrated with 0.25% bupivacaine in sterile saline post-surgery and closed in layers using 5-0 monofilament nylon sutures. The surgical procedure lasted approximately 10 min, after which mice were returned to the anesthesia chamber to complete a 2 h isoflurane exposure. During recovery, animals were placed on a temperature-controlled pad for 30–60 min to stabilize their core temperature. Postoperative monitoring included continuous observation for 4 h immediately following anesthesia/surgery, with daily assessments thereafter. Sham mice were transported to the surgical suite but did not receive anesthesia or surgery, and they remained in their home cages under room air exposure for 2 h, reflecting clinically relevant conditions. Blinding of surgical personnel was not feasible due to visible abdominal incisions; however, investigators performing tissue collection and data analysis were blinded to the age group and treatment condition.

For long-term assessment of cardiac tissue, a separate cohort of 20-month-old mice were subjected to laparotomy and 2 h isoflurane exposure as described in a previous publication [25]. For the GA-alone control group, a cohort of 20-month-old mice underwent 2 h isoflurane exposure without surgery. In brief, following a battery of neurological behavioral tests that were conducted for up to 38 days, mice were euthanized at 5 weeks after cessation and the hearts were obtained for downstream molecular experiments. For this section, there were a total of three experimental groups: 20-month Sham (*n* = 5), 20-month Iso (*n* = 5), 20-month Iso/Op (*n* = 5).

### 2.2. RNA Extraction and Bulk RNA Sequencing (RNA-Seq)

Following euthanasia at 24 h and 5 weeks post-Iso/Op, mice were perfused with 50 mL of ice-cold saline, and the entire heart was dissected for downstream processing. The dissected cardiac tissue was homogenized in TRIzol reagent (Thermo Fisher Scientific, Waltham, MA, USA) following the manufacturer’s instructions. Using the homogenate, total RNA was extracted from the heart of both sham and Iso/Op mice using the Direct-zol™ RNA Miniprep Plus kit (Cat# R2073-A, Zymo Research, Irvine, CA, USA). Extracted RNA samples were sent to Psomagen (Rockville, MD, USA) for mRNA library preparation via Illumina Stranded Total RNA Prep, Ligation with Ribo-Zero Plus kit (Cat# 20040529, Illumina, San Diego, CA, USA). A total of 100 ng RNA per sample was used for library preparation, which produced 150 bp paired-end reads on a NovaSeq X Plus 25B platform (Illumina, San Diego, CA, USA).

### 2.3. Statistical and RNA-Seq Analysis

RNA-seq reads were aligned to the mouse reference genome (GRCm39) using STAR aligner (v 2.7.5) [29], with GENCODE gene annotation (version M33). Gene and isoform expression levels were quantified as transcripts per million (TPM) using RSEM 1.3.3 with -max_frag_len 1000 as the maximum fragment length parameter, and otherwise default parameters [30]. TPM values were used for visualization and comparative expression plots, whereas DESeq2-normalized counts were used for differential expression testing. Differential expression analysis was conducted using DESeq2 version 1.42.1 with false discovery rates (FDR) calculated via the Benjamini–Hochberg method. Genes with FDR <0.05 were considered differentially expressed and subjected to downstream pathway enrichment analysis. Gene ontology and pathway analyses were performed using clusterProfiler 4.10.1 [31] on the DESeq2 output. We used the genome-wide mouse annotation database package from R/Bioconductor (org.Mm.eg.db, version 3.21.0) [32] for the Gene Ontology analysis. Detailed statistical analyses for each assay are provided in the figure legends, with a significance threshold set at *p* < 0.05.

## 3. Results

### 3.1. Short-Term Iso/Op Exposure Disrupts Cardiac Fatty Acid Metabolism and Cytoskeletal Gene Expression in Young Adult Mice 24 h Post-Exposure

**Figure 1:**
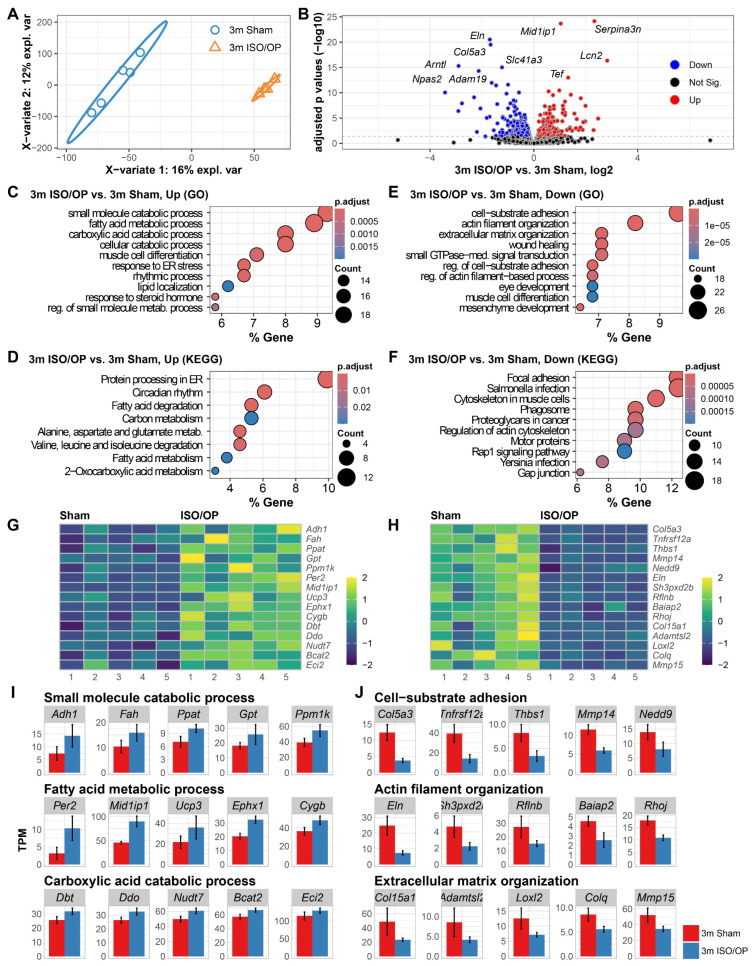
Iso/Op drives acute alterations in transcriptomic profile of cardiac tissue in young adult mice at 24 h after isoflurane (ISO) exposure. (**A**) PLSDA plot for all normalized transcriptome genes is depicted for young adult 3-month-old (3 m) mice with or without ISO exposure following laparotomy. (**B**) Volcano plot displaying differentially expressed genes (DEGs) in the 3m Iso/Op vs. 3m Sham comparison. (**C**,**D**) Pathway enrichment analysis of upregulated DEGs with Gene Ontology molecular processes (**C**) and KEGG pathways (**D**). (**E**,**F**) Pathway enrichment analysis of downregulated DEGs with Gene Ontology molecular processes (**E**) and KEGG pathways (**F**). Z-score normalization was applied to gene expression values. Heatmaps illustrate sample-wise variation (columns) across genes (rows). Color intensity reflects relative transcript abundance across samples: warmer colors for higher, and cooler for lower. (**G**–**J**) Heatmap and bar graph of genes involved in the top three enriched pathways. *n* = 5 mice/group.

To evaluate the cardiac transcriptional effects of short-term isoflurane-based perioperative stress in young adult mice, we subjected 3-month-old (approximately equivalent to 20–30 human years of age) C57BL/6 male mice to laparotomy and 2 h Iso exposure. Cardiac transcriptomic profiling at 24 h post-exposure revealed a clear separation between Sham and Iso/Op groups by partial least squares discriminant analysis (PLSDA, Figure 1A). Following pairwise comparison, differentially expressed genes (DEGs) identified in the volcano plot showed a comparable number of up- and downregulated transcripts in the hearts of Iso/Op mice (Figure 1B), with the mostly highly ranked DEGs including both upregulated (*Serpina3n*, *Mid1ip1*, *Lcn2*, and *Tef*) and downregulated (*Eln*, *Col5a3*, *Arntl*, *Npas2*, *Slc41a3*, and *Adam19*) genes. Gene ontology (GO) enrichment analysis of upregulated DEGs revealed significant overrepresentation of small molecule and fatty acid catabolic processes (Figure 1C). Consistent with GO findings, Kyoto Encyclopedia of Genes and Genomes (KEGG) pathway analysis identified enrichment of “protein processing in ER”, “circadian rhythm”, and “fatty acid degradation” pathways (Figure 1D). In contrast, downregulated DEGs were enriched for GO terms related to “cell-substrate adhesion”, “actin filament organization”, and “extracellular matrix organization” (Figure 1E). Moreover, further analysis revealed that “focal adhesion”, “salmonella infection”, and “cytoskeleton in muscle cells” were the most significantly enriched pathways by KEGG analysis (Figure 1F). Representative genes from the most significantly enriched GO processes were visualized as heatmaps for up- and downregulated DEGs (Figure 1G,H). To facilitate quantitative comparison, normalized transcript abundance (TPM) of representative genes was plotted as bar graphs, accounting for library size and sequencing depth (Figure 1I).

### 3.2. Middle-Aged Mice Exhibit Impaired Fatty Acid Metabolism and Muscle System Processes 24 h After Cessation

**Figure 2:**
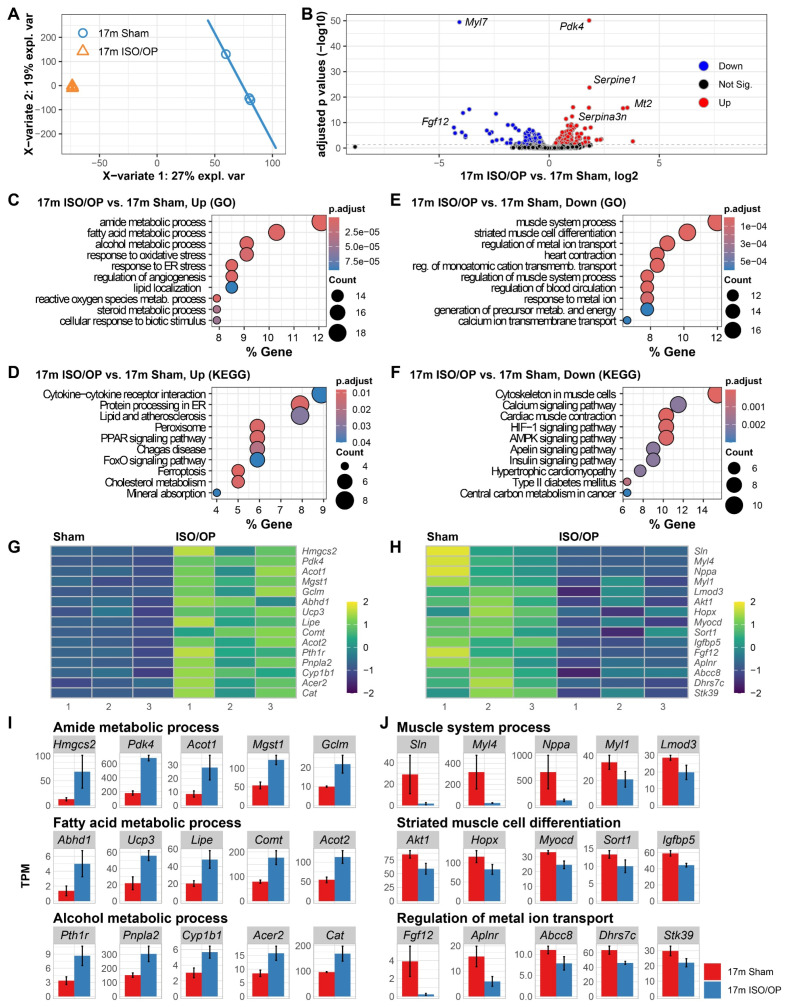
Heart transcriptomic profiling at 24 h after short-term Iso/Op reveals altered metabolic and muscle cell pathways in aged 17-month-old (17 m) mice. (**A**) PLSDA showing clear separation of heart transcriptomes across groups. (**B**) Volcano plot of differentially expressed genes (DEGs) comparing 17m Iso/Op vs. 17m Sham. (**C**,**D**) Enriched GO molecular processes (**C**) and KEGG pathways (**D**) for upregulated DEGs. (**E**,**F**) Enriched GO molecular processes (**E**) and KEGG pathways (**F**) for downregulated DEGs. Heatmaps of representative genes contributing to the top enriched GO term pathways (**G**,**H**). (**I**,**J**) Bar plots illustrating expression of selected DEGs linked to the top three enriched pathways in up- (**I**) and down- (**J**) regulated DEGs. *n* = 3 mice/group.

We next examined the effects that Iso/Op may have on 17-month old mice, which are the equivalent of 51–54 years of age for humans [33,34], a late middle-aged to old phase. At 24 h post-exposure, PLSDA revealed that Iso/Op accounted for 27% of the observed transcriptional variance (Figure 2A), whereas pairwise comparison showed a significant number of total DEGs (Figure 2B), the most notable ones consisting of four upregulated genes (*Pdk4*, *Serpine1*, *Mt2*, *Serpina3n*), along with two downregulated ones (*Myl7*, *Fgf12*). GO enrichment analysis of upregulated DEGs demonstrated heightened activity in “amide metabolic process”, “fatty acid metabolic process”, and “alcohol metabolic process” (Figure 2C). In KEGG terms, we found a coordinated upregulation of genes related to “cytokine-cytokine receptor interaction”, “protein processing in ER”, and “lipid and atherosclerosis” processes (Figure 2D). In contrast, downregulated DEGs were found to encompass “muscle system process”, “striated muscle cell differentiation”, and “regulation of metal ion transport” in GO molecular processes, suggesting that Iso/Op affected muscle cells in the heart at 24 h after cessation (Figure 2E). Moreover, KEGG pathways revealed that genes involved in “cytoskeleton in muscle cells”, “calcium signaling pathway”, and “cardiac muscle contraction” were downregulated (Figure 2F). To visualize the expression patterns driving the GO biological processes, we generated heatmaps of the key genes belonging to these pathways (Figure 2G,H), and we quantified and visualized as bar graphs the normalized transcript abundance (TPM) of representative DEGs within each GO category, adjusted for library size and sequencing depth (Figure 2I).

### 3.3. Old Mice Exposed to Iso Exhibit Upregulation of Fatty Acid Metabolic Processes and Downregulation of Muscle Cell Differentiation

**Figure 3:**
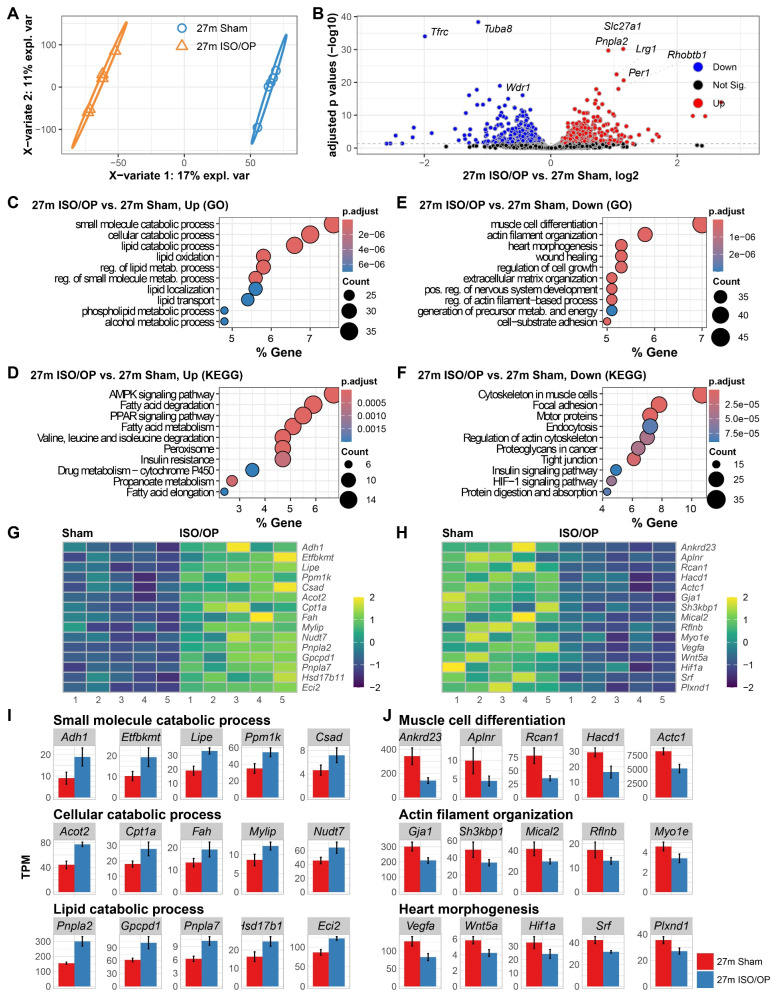
Short-term isoflurane (Iso) exposure alters acute transcriptomic responses in the heart of aged 27-month-old (27 m) mice 24 h after cessation. (**A**) PLSDA of heart transcriptomes across groups shows clear separation. (**B**) Volcano plot of differentially expressed genes (DEGs) comparing 27m Iso/Op vs. 27m Sham. (**C**,**D**) Enriched GO molecular processes (**C**) and KEGG pathways (**D**) for upregulated DEGs. (**E**,**F**) Enriched GO molecular processes (**E**) and KEGG pathways (**F**) for downregulated DEGs. Heatmaps of representative genes contributing to the top enriched GO term pathways (**G**,**H**). (**I**,**J**) Bar plots illustrating expression of selected DEGs linked to the top three enriched pathways in up- (**I**) and down- (**J**) regulated DEGs. *n* = 5 mice/group.

After exploring the effects of Iso/Op on the cardiovascular system of young and late middle-aged mice, we examined the effects this procedure may have on old 27-month-old mice, approximately equivalent to 80 years of age in humans. PLSDA of all normalized genes at 24 h after cessation showed a clear separation between sham and Iso/Op mice, with the procedure contributing to 17% of the variation between groups (Figure 3A). In addition, DEGs between groups showed a robust change in the cardiac transcriptomic profile (Figure 3B), with notable upregulation observed in *Slc27a1*, *Pnpla2*, *Lrg1*, *Rhobtb1*, and *Per1*, which span core aspects of cardiac physiology, including energy metabolism (*Slc27a1* and *Pnpla2*), fibrosis regulation (*Lrg1*), vascular tone maintenance (*Rhobtb1*), and hemodynamic circadian modulation, respectively (*Per1*). In addition, the three notable downregulated genes, *Tfrc*, *Tuba8*, and *Wdr1*, have been reported to be involved in iron metabolism, cytoskeletal dynamics, and actin filament regulation, respectively, which are critical to cardiac cell physiology.

To extract biological meaning from the list of regulated genes, we performed a functional enrichment analysis using GO molecular process and KEGG pathway databases. Pathway enrichment analysis of the upregulated DEGs showed “small molecule catabolic”, “cellular catabolic”, and “lipid catabolic” as the top GO term processes being mediated by the Iso/Op treatment (Figure 3C). In KEGG analysis, “AMPK signaling pathway”, “fatty acid degradation”, and “PPAR signaling pathway” were the top enriched pathways (Figure 3D). Conversely, pathways involved in “muscle cell differentiation”, “actin filament organization”, and “heart morphogenesis” were highly prominent among the downregulated DEGs (Figure 3E), while KEGG analysis revealed a strong signature in genes involved with “cytoskeleton in muscle cells”, “focal adhesion”, and “motor proteins” (Figure 3F). Finally, we visualized the expression patterns of key genes from the enriched GO processes using heatmaps (Figure 3G,H), and we quantified the TPM of representative DEGs from each category using bar graphs, adjusted for the library size and sequencing depth (Figure 3I).

### 3.4. The Acute Cardiac Transcriptional Response to Iso/Op Is Age-Dependent After 2 h of Exposure

**Figure 4:**
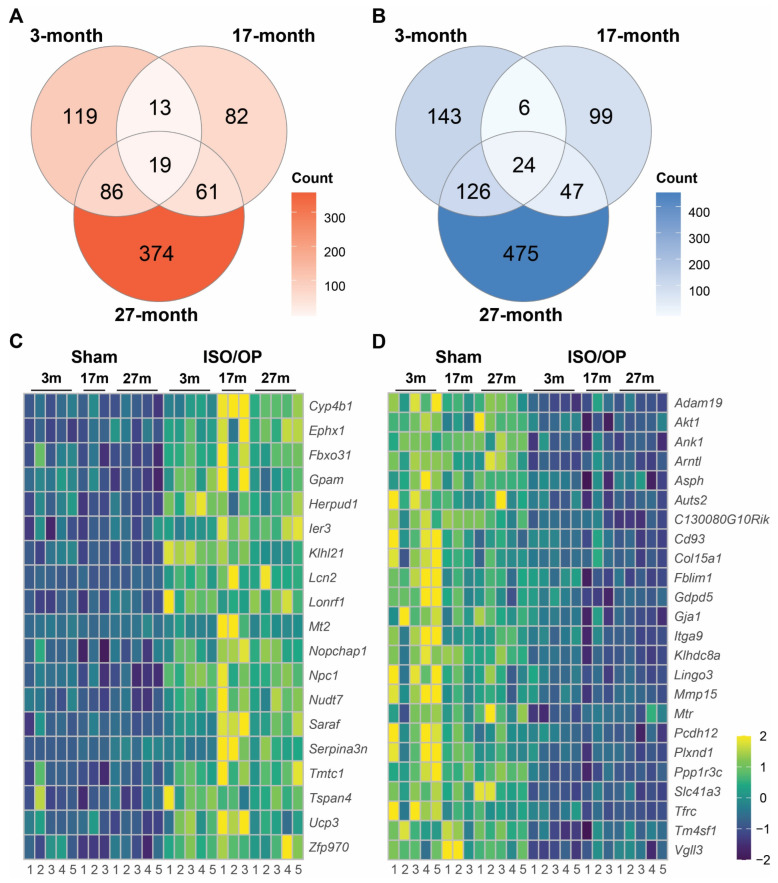
Overlap analysis of differentially expressed genes (DEGs) across different age groups identifying key genes. (**A**,**B**) Venn diagrams of the up- (**A**) and down- (**B**) regulated DEGs obtained from Iso/Op vs. Sham comparison across different age groups. (**C**,**D**) Heatmaps of overlapping genes in up- (**C**) and down- (**D**) regulated gene sets. *n* = 3–5 mice/group.

We next explored whether key Iso/Op-mediated targets could be isolated from across different age groups by examination of overlapping genes. Amongst the upregulated DEGs derived from 3 m, 17 m, and 27 m mice, a total of 19 genes showed overlap in the Iso/Op vs. sham mice comparison groups (Figure 4A). The expression patterns of these key genes across the three different age groups were visualized as a heatmap following normalization by their respective sham groups (Figure 4B). Several shared DEGs have established roles in cardiac stress and aging, including *Cyp4b1*, *Ephx1*, *Fbxo31*, and *Lcn2*, which have been implicated in hypertrophy, ischemic recovery, endothelial senescence, and inflammatory cardiomyocyte injury, respectively [35,36,37,38,39]. Overall, these results suggest that acute Iso/Op in aged mice induces an upregulation of genes related to oxidative stress, Ca^2+^ handling, hypertrophy regulation, and lipid metabolism, which seem to increase with age.

Next, we examined the overlapping genes derived from downregulated DEGs, which yielded a total of 24 genes (Figure 4C), visualized as heatmaps to explore their changes across the three different age groups (Figure 4D). Key genes encompassed in these processes include *Akt1*, *Asph*, *Mtr*, *Tfrc*, *Pcdh12*, and *Ppp1r3c*, which have been identified to play distinctive roles in cardiac function by regulating energy homeostasis, calcium dynamics, and metabolism. Moreover, the gene products of *Adam19*, *Akt1*, *Arntl*, and *Cd93* have been reported to modulate cellular senescence and cardiac aging [40,41,42,43]. Together, these findings highlight that Iso/Op exposure elicits convergent transcriptional responses across ages, with fatty acid metabolism and energy regulatory pathways, along with dysregulation of genes involved in cellular senescence, emerging as core molecular signatures of cardiac stress at 24 h post-exposure.

### 3.5. Iso/Op Treatment Induces Long-Term Dysregulation of Diabetic Cardiomyopathy-Associated Genes in Aged Mice

A previous study from our group demonstrated that Iso/Op exposure in 20-month-old mice induces persistent olfactory dysfunction, reduced limb strength, and impaired motor coordination indicative of frailty, accompanied by delayed cognitive deficits and increased apathy in the same cohort [25]. Emerging evidence and scientific statements from the American Heart Association have shown unequivocally that the brain and heart are interdependent organ systems with intricate pathogenic mechanisms that link heart conditions with microstructural changes in the brain [26,27,28]. Building on these findings, we subsequently collected heart tissues from the same batch of geriatric mice at 5 w after cessation to investigate the long-term cardiovascular effects of Iso/Op.

**Figure 5:**
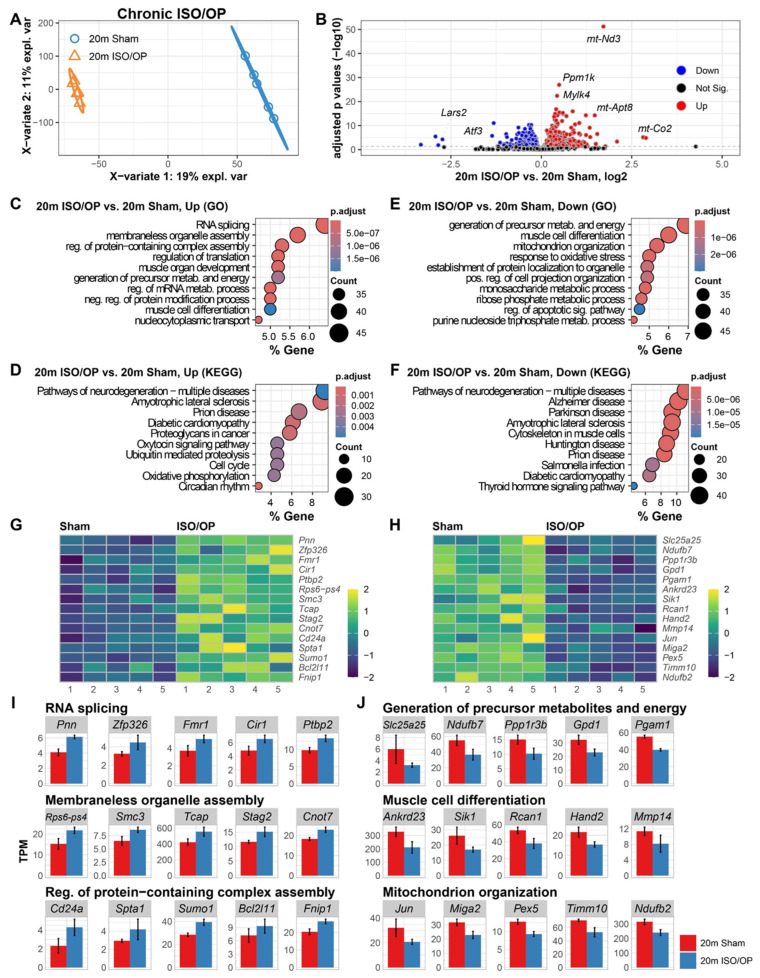
Chronic cardiac transcriptomes at 5 weeks post-cessation reveal persistent alterations to cellular assembly and energy homeostasis in aged mice. (**A**) Multivariate analysis (PLSDA) reveals persistent divergence of cardiac transcriptomes induced by laparotomy and isoflurane exposure (Iso/Op). (**B**) Volcano plot of differentially expressed genes (DEGs) comparing 20m Iso/Op vs. 20m Sham. (**C**,**D**) Enriched GO molecular processes (**C**) and KEGG pathways (**D**) for upregulated DEGs. (**E**,**F**) Enriched GO molecular processes (**E**) and KEGG pathways (**F**) for downregulated DEGs. Heatmaps of representative genes contributing to the top enriched GO term pathways (**G**,**H**). (**I**,**J**) Bar plots illustrating expression of selected DEGs linked to the top three enriched pathways in up- (**I**) and down- (**J**) regulated DEGs. *n* = 5 mice/group.

To assess long-term cardiac remodeling, RNA-seq was performed on heart tissue collected five weeks after Iso/Op exposure in 20-month-old mice. Initial analysis of the cardiac transcriptional profiles showed a clear separation between the Sham and Iso/Op mice by PLSDA, with the surgery contributing to 19% of the total variance (Figure 5A). Pairwise comparisons further identified distinct molecular alterations, with genes such as *Lars2* and *Atf3* showing marked downregulation, while *mt-Nd3*, *Ppm1k*, *Mylk4*, *mt-Atp8*, and *mt-Co2* were significantly upregulated in Iso/Op-exposed mice (Figure 5B). To interpret the biological implications of the upregulated DEGs, we performed GO enrichment analysis. This revealed a strong signature of genes involved in “RNA splicing”, “membraneless organelle assembly”, and “regulation of protein-containing complex assembly” (Figure 5C). Consistent with these findings, KEGG pathway analysis highlighted a significant enrichment for pathways associated with neurodegeneration, including “amyotrophic lateral sclerosis”, “prion disease”, “diabetic cardiomyopathy”, and “proteoglycans in cancer” (Figure 5D). Meanwhile, the top downregulated pathways consisted of “generation of precursor metabolism and energy”, “muscle cell differentiation”, and “mitochondrion organization” among the GO terms (Figure 5E), while KEGG analysis also showed a strong signature in neurodegeneration, with Alzheimer’s and Parkinson’s diseases as the next most enriched pathways (Figure 5F). To further illustrate these transcriptional shifts, we visualized the expression patterns of key genes within enriched GO categories as heatmaps (Figure 5G,H) and quantified representative DEGs from each pathway as normalized TPM bar plots adjusted for library size and sequencing depth (Figure 5I).

**Figure 6:**
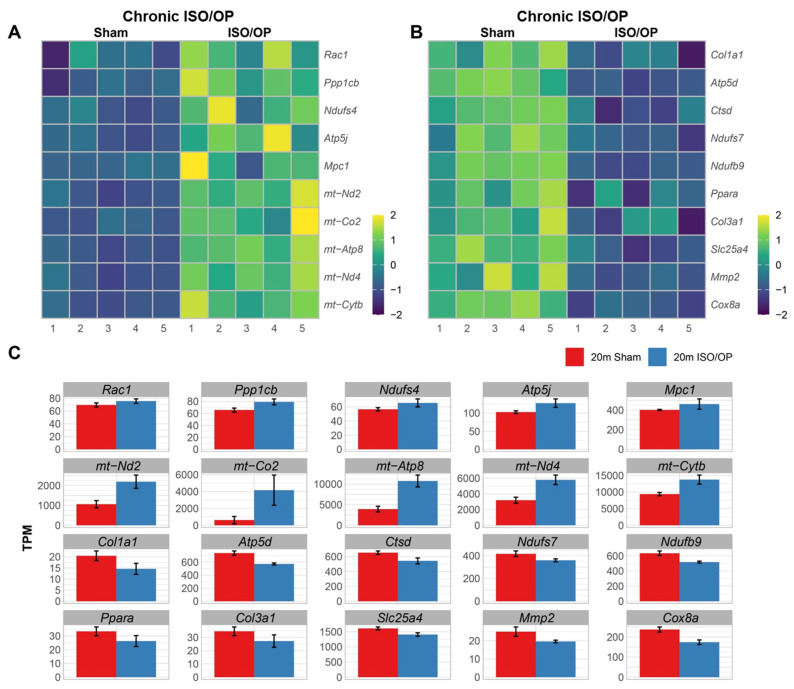
Chronic cardiac transcriptomes at 5 weeks post-cessation show alterations to diabetic cardiomyopathy genes in aged mice. (**A**,**B**) Heatmaps depict expression patterns of the up- (**A**) and down- (**B**) regulated genes involved with cardiomyopathy, which illustrates sample-wise variation (columns) across genes (rows). Z-score normalization was applied to gene expression values. Color intensity reflects relative transcript abundance across samples, with warmer colors for higher, and cooler for lower. Heatmap and bar graph of genes involved in the top three enriched pathways. (**C**) Bar graph of the genes related to cardiomyopathy. *n* = 5 mice/group.

In the KEGG pathway analysis for upregulated DEGs, the top three enrichments were related to neurodegeneration and neuroinflammation, but the fourth term was diabetic cardiomyopathy (CM), which was specific to cardiac tissue and needed further interrogation. Notable genes that mediate this pathological process include *Rac1*, *Ppp1cb*, *Ndufs4*, *Atp5j*, and *Mpc1*, along with the mitochondrial DNA genes *mt-Nd2*, *mt-Co2*, *mt-Atp8*, *mt-Nd4*, and *mt-Cytb*, which were upregulated in the Iso/Op mice when compared to sham animals (Figure 6A). In contrast, downregulated DEGs that overlap with diabetic cardiomyopathy included *Col1a1*, *Atp5d*, *Ctsd*, *Ndufs7*, *Ndufb9*, *Ppara*, *Col3a1*, *Slc25a4*, *Mmp2*, and *Cox8a* (Figure 6B). For rigorous quantification, gene-level expression was depicted as bar graphs with normalized TPM plotted on the y-axis (Figure 6C). Collectively, these findings reveal that Iso/Op induces persistent, transcriptome-level remodeling of the aged heart, marked by mitochondrial and metabolic dysregulation consistent with diabetic cardiomyopathy and neurodegenerative signaling pathways.

**Figure 7:**
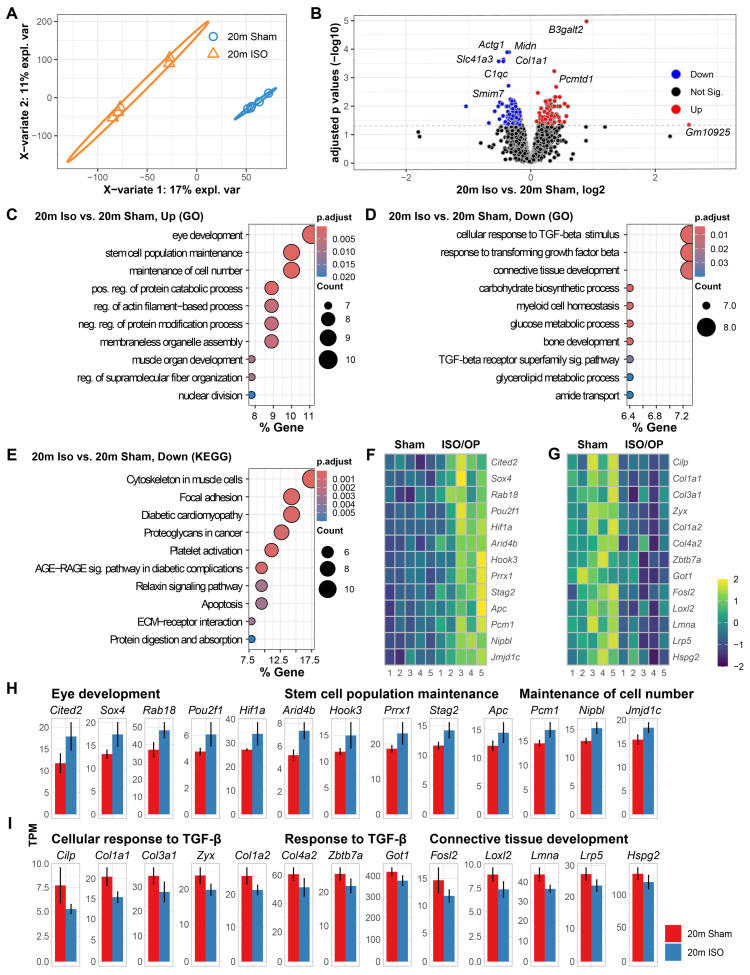
RNA sequencing at 5 weeks after exposure to isoflurane (ISO) alone reveals persistent alterations to the cardiac transcriptomic profile of aged mice. (**A**) PLSDA shows clear divergence of cardiac transcriptomes induced by 2 h isoflurane exposure (ISO). (**B**) Volcano plot of differentially expressed genes (DEGs) comparing 20 m ISO vs. 20 m Sham. (**C**,**D**) Enriched GO molecular processes for up- (**C**) and down- (**D**) regulated DEGs. (**E**) Enriched KEGG pathways for downregulated DEGs. (**F**,**G**) Heatmaps of representative genes contributing to the top enriched GO term pathways. (**H**,**I**) Bar plots illustrating expression of selected DEGs linked to the top three enriched pathways in up- (**H**) and down- (**I**) regulated DEGs. *n* = 5 mice/group.

To distinguish the relative contribution of isoflurane from surgical stress, we analyzed cardiac transcriptomes from age-matched mice exposed to isoflurane alone [25]. PLSDA of the cardiac transcriptomes revealed a distinct separation between sham and Iso mice at 5 w after cessation, with 17% of the total variance being found attributable to GA exposure (Figure 7A). Pairwise comparisons further identified distinct molecular alterations, with genes such as *Actg1*, *Midn*, *Slc41a3*, *Col1a1*, *C1qc*, and *Smim7* showing marked downregulation, while *B3galt2* and *Pcmtd1* were significantly upregulated in the 20-month-old mice that were exposed to Iso for 2 h (Figure 7B). Afterwards, enrichment analysis of the upregulated DEGs was employed to identify GO terms that were over-represented. GO terminology showed significant enrichment in genes that were related to “eye development”, “stem cell population maintenance”, and “maintenance of cell number” processes (Figure 7C). On the other hand, downregulated DEGs were shown to be enriched in genes that mediate “cellular response to TGF-beta stimulus”, “response to transforming growth factor beta”, and “connective tissue development” (Figure 7D). Complementing this, KEGG pathway analysis demonstrated significant enrichment in “cytoskeleton in muscle cells”, “focal adhesion”, and “diabetic cardiomyopathy” pathways amongst the DEGs that were downregulated by Iso exposure (Figure 7E). Key genes derived from the top three up- and downregulated pathways were visualized as a heatmap to further explore their long-term expression patterns in the hearts at 5 w after cessation (Figure 7F,G), while quantification of the normalized TPM values was represented as bar graphs for each gene (Figure 7H,I).

## 4. Discussion

In this study, we demonstrate that even brief exposure to isoflurane-based perioperative stress induces age-dependent transcriptional changes in the heart, revealing a previously underappreciated cardiac sensitivity to anesthesia and surgery. In young adult mice, Iso/Op acutely altered fatty acid metabolism and cytoskeletal organization, consistent with early metabolic stress and transient structural remodeling. With advancing age, these transcriptional responses intensified and diversified: 17-month-old mice exhibited suppression of muscle system and calcium signaling pathways, whereas aged mice displayed enhanced lipid catabolism, activation of PPAR and AMPK signaling, and downregulation of pathways governing contractility and morphogenesis. Across all ages, upregulation of fatty acid metabolism and oxidative stress genes suggests metabolic stress as a core acute response. At the chronic timepoint, aged mice failed to fully resolve these transcriptional changes, retaining signatures linked to mitochondrial dysfunction and diabetic cardiomyopathy, including after isoflurane exposure alone. These findings position Iso as a driver of age-escalating cardiac metabolic and structural stress, providing a potential mechanistic link between perioperative exposure, energy dysregulation, and increased cardiovascular vulnerability in older patients.

In young adult hearts, Iso/Op exposure triggered acute transcriptional changes involving fatty acid metabolism, ER-associated protein processing, and cytoskeletal organization. Because the myocardium derives the majority of its ATP from fatty acid oxidation (FAO) under physiological conditions [44], FAO-associated transcripts suggest acute energetic stress. Enrichment of ER protein processing-related transcripts suggests that perioperative exposure imposes a temporary proteostatic load, consistent with prior evidence that volatile anesthetics, such as Iso, disrupt mitochondrial function and activate ER stress pathways in the CNS [12,13,45]. In addition, studies also show that Iso can destabilize the actin cytoskeleton and weaken integrin-mediated adhesion [46,47,48], which may reflect a broader cellular vulnerability to structural stress. While our RNA-seq data cannot resolve these mechanisms directly, the transcriptional profile observed here aligns with a pattern of transient metabolic and proteostatic challenge, emphasizing that even brief anesthesia and surgical stress can perturb cardiomyocyte homeostasis.

At 24 h post-Iso/Op, converging DEGs across ages indicate disrupted fatty acid metabolism and energy regulation, consistent with age-related declines in mitochondrial β-oxidation and lipid accumulation [22,24]. Isoflurane has been reported to induce ER stress characterized by CHOP and caspase-12 activation and AMPK-linked autophagy [11,12,49]. Although pathway enrichment was not significant, several DEGs are known to participate in cardiac aging processes. Aging further amplifies metabolic imbalance through impaired mitophagy, reduced chaperone function, and baseline SASP accumulation [24,50]. Additionally, Iso-induced cytoskeletal disruption via p75NTR–RhoA signaling has been shown to impair mechanotransduction and homeostasis [51]. Together, Iso/Op provokes a multifaceted stress response, metabolic dysfunction, ER stress, and senescence-related gene activation, exacerbated in aged myocardium with diminished compensatory capacity.

The 5-week post-Iso/Op timepoint was selected to capture a delayed phase of postoperative vulnerability previously identified in aged mice. Prior studies have shown that 20-month-old mice exposed to isoflurane and surgery develop persistent olfactory deficits, reduced strength and motor coordination indicative of frailty, and delayed cognitive impairments affecting learning, memory, and motivation. Profiling cardiac transcription at this interval therefore enabled the assessment of whether ongoing neurological dysfunction and functional decline are accompanied by sustained cardiac molecular remodeling. Persistent cardiac transcriptional changes during this phase support the possibility of coordinated heart–brain interactions following anesthesia and surgical stress, consistent with emerging evidence in aging and neurodegenerative disorders. At 5 weeks post-Iso/Op, the cardiac transcriptome shifts from acute stress responses to chronic structural and metabolic remodeling. Upregulation of RNA splicing and processing genes suggests ongoing transcriptional reprogramming, a feature commonly observed in pathological cardiac remodeling, where altered splicing of sarcomeric and calcium-handling proteins impairs contractility and metabolism [52,53]. Concurrent enrichment of diabetic cardiomyopathy-like signatures points to sustained metabolic stress and disrupted mitochondrial energetics, while the downregulation of anabolic and mitochondrial organization pathways reflects declining bioenergetic efficiency [54]. Overall, our findings suggest that a single short-term exposure to Iso/Op can drive maladaptive remodeling long after its cessation, which is marked by aberrant splicing, mitochondrial disarray, and hypometabolic degeneration, akin to late-stage heart failure.

Finally, by isolating chronic-timepoint DEGs related to diabetic CM, we found many upregulated mitochondrial genes associated with hyperglycemia-driven dysfunction. Iso exposure has been shown to induce transient hyperglycemia by impairing insulin signaling and glucose uptake, as shown in rodent, canine, and human studies [55,56,57,58]. Chronic hyperglycemia also triggers ER stress, a hallmark of diabetic CM, by overwhelming the protein-folding capacity and activating maladaptive UPR signaling [59]. Prolonged ER stress disrupts calcium balance, elevates oxidative stress, and promotes mitochondrial dysfunction and apoptosis [60,61,62]. In parallel, oxidative stress can further exacerbate mitochondrial damage and impair metabolic flexibility, reinforcing transcriptional programs associated with diabetic cardiomyopathy even in the absence of overt structural injury [63,64]. Together with dysregulated lipid catabolism observed acutely, these data suggest ER stress is a key mediator linking Iso-induced metabolic overload to persistent diabetic-like cardiac gene signatures.

In our study, the RNA sequencing of heart tissues of our Iso/Op mouse model was conducted at 24 h and 5 weeks post Iso exposure. The RNA sequencing identified Iso/Op DEGs associated with fatty acid metabolic process, carbon metabolism, and cardiomyocyte functions. One of the upregulated genes is the one encoding pyruvate dehydrogenase kinase 4 (Pdk4). Pdk4 is a key regulator of glucose metabolism in the heart, and high Pdk4 expression interrupts cardiac glucose metabolism and is associated with interruption of fatty acid metabolism [40,65]. Another Iso-upregulated gene, *Serpine 1*, is known to be strongly associated with cardiovascular diseases, such as atherosclerosis and heart failure [66,67]. High expression of Serpine1 contributes to cardiomyocyte apoptosis in sepsis [68]. Furthermore, Iso exposure suppresses transferrin receptor (Tfrc) expression in the heart. Tfrc is responsible for iron transportation. It is critical for iron homeostasis-mediated regulation of heart function. Lack of Tfrc causes iron deficiency and is associated with cardiomyopathy and heart failure [69]. In addition, Iso exposure may increase the risk of impaired cytoskeleton function by suppressing *Tub8a* gene expression. *Tuba8* encodes an alpha-tubulin isoform expressed in heart tissue. It contributes to microtubule organization, structural integrity and contractile function of cardiomyocytes. Iso exposure-mediated inhibition of *Tuba8* expression may lead to altered cardiomyocyte contractility.

Together with prior studies on GA/Op-induced neurological deficits, our findings suggest that acute perioperative transcriptomic changes may signal long-term cardiac vulnerability and contribute to delayed brain dysfunction in aged mice [25]. Chronic β-oxidation suppression and lipid accumulation can cause mitochondrial and ER stress, driving insulin resistance and proteostatic failure, hallmarks of diabetic cardiomyopathy [70,71]. Similar metabolic and oxidative disruptions occur in the brain after anesthesia, driving synaptic remodeling and cognitive decline [72,73,74]. These shared transcriptomic patterns between the heart and brain point to coupled dysregulation of fatty acid metabolism, redox balance, and ER stress. Mitochondrial dysfunction in the heart can release inflammatory mediators that modulate neuroinflammation [75,76,77], while neural mitochondrial injury may impair autonomic regulation of cardiac function, forming a bidirectional feedback loop [78,79,80].

Possible limitations of this study include the absence of female mice. Prior work shows sex differences in cardiac function and pathology [81,82,83], as well as context- and species-dependent sex effects on neurological outcomes after GA/OP [84,85,86,87]. Clinical studies suggest that males are more prone to postoperative delirium and cognitive dysfunction [88], whereas preclinical findings vary, including sex-specific effects in newborn rats and reversed patterns in Alzheimer’s disease mouse models [89]. Together, these observations highlight potential sex dimorphism in postoperative recovery that warrants further investigation. Another limitation is the absence of anesthesia-only and acute perioperative controls across all age groups, which limits full separation of anesthesia-versus surgery-specific transcriptional effects. However, an isoflurane-only control was included in the 20-month cohort, and prior studies in aged mice show largely transient effects at the delayed collection timepoint. Given the limited availability of aged mice, we prioritized long-term remodeling under the clinically relevant combined isoflurane-plus-surgery condition and minimized animal use by not adding acute arms. An important question raised by this work is whether Iso/Op-induced cardiac transcriptional changes are reversible. Across the timepoints examined, we observed no evidence of normalization in aged hearts; instead, pathways related to mitochondrial dysfunction, oxidative stress, and metabolic remodeling persisted or intensified at 5 weeks post-operation. This pattern suggests sustained or progressive remodeling rather than recovery, consistent with age-related impairments in stress resolution. Although longer-term outcomes cannot be determined here, the data indicate that Iso/Op can induce durable cardiac remodeling in aged mice over clinically relevant timescales.

A key limitation of this study is that bulk RNA-seq offers a descriptive view of postoperative transcriptional remodeling without direct assessment of cardiac function or oxidative stress. Consequently, the physiological significance of the observed gene expression changes cannot be conclusively determined from the present data alone. However, perioperative myocardial infarction and mortality are well-recognized clinical complications for elderly patients [90,91], which arise from the interaction of surgical stress, patient comorbidities, and anesthetic management, underscoring the heart as a vulnerable target in the perioperative period. Within this established clinical context, the transcriptomic alterations identified here may reflect molecular correlates of cardiac stress or maladaptive remodeling that precede overt functional decline. Nonetheless, future studies integrating functional cardiac assessments and biochemical markers of oxidative injury will be essential to establish causal links between these molecular signatures and postoperative cardiac outcomes.

Dysregulated heart function is increasingly recognized as a contributor to neurodegenerative diseases like Alzheimer’s and Parkinson’s [92,93,94,95]. Evidence links open-heart surgery to postoperative cognitive dysfunction (POCD) [96,97,98], with one study reporting elevated CSF Abeta after cardiopulmonary bypass [99]. Few studies have examined GA’s effects on the cardiovascular system outside of heart surgery [100]. To our knowledge, this study is among the first to report subtle molecular changes in cardiac tissue following Iso/Op. Our findings show elevated cardiac ER stress 24 h post-surgery, a key perioperative window. Integrating cardiac transcriptomics from 5-week awake mice with prior observations of neural deficits offers insight into how the heart–brain axis drives neurological consequences after Iso/Op in aged mice. Overall, cardiac ER stress may be a potential mechanism and target for future studies on POCD and postoperative delirium.

From a clinical perspective, anesthesia is an unavoidable component of modern surgical care, particularly in aging populations. However, anesthetic choice, perioperative management, and postoperative monitoring remain modifiable factors. Our findings suggest that isoflurane exposure combined with operative stress can induce age-dependent cardiac transcriptional remodeling, raising the possibility that older patients may experience persistent molecular alterations even after uneventful noncardiac surgery. While the functional consequences of these changes remain to be fully defined, they highlight a potential biological substrate for delayed or subclinical cardiovascular vulnerability in the elderly. These data provide a rationale for future studies aimed at optimizing anesthetic strategies and identifying perioperative biomarkers of cardiac risk in aging and geriatric patients.

## 5. Conclusions

The present data demonstrates that Iso-based perioperative exposure elicits pronounced age-dependent acute transcriptional responses in the heart, with young adult (3 m), late middle-aged (17 m), and old (27 m) mice exhibiting distinct patterns of metabolic, calcium-handling, and structural pathway disruption within 24 h of Iso/Op. These acute responses were progressively amplified with age, shifting from primarily metabolic and cytoskeletal perturbations in young hearts to coordinated suppression of contractile and morphogenetic programs alongside maladaptive lipid and mitochondrial remodeling in older hearts. Importantly, longitudinal analysis revealed that a subset of these stress-induced programs persisted well beyond the acute phase, as 20 m mice displayed sustained transcriptomic remodeling five weeks after Iso/Op, driven predominantly by Iso exposure and characterized by mitochondrial dysfunction, extracellular matrix remodeling, and diabetic cardiomyopathy-associated gene signatures. Together, these findings indicate that perioperative Iso exposure can trigger age-amplified acute cardiac stress responses that, in aging hearts, transition into durable metabolic and structural reprogramming, implicating impaired lipid utilization and mitochondrial homeostasis as potential mechanisms of long-term cardiovascular vulnerability.

## Data Availability

The original contributions presented in this study are included in the article. Further inquiries can be directed to the corresponding author(s).

## Abbreviations

AMPK	AMP-activated protein kinase
ATP	Adenosine triphosphate
CNS	Central nervous system
DEGs	Differentially expressed genes
ER	Endoplasmic reticulum
FAO	Fatty acid oxidation
GA	General anesthesia
GO	Gene ontology
Iso	Isoflurane
KEGG	Kyoto Encyclopedia of Genes and Genomes
NAD	Nicotinamide adenine dinucleotide
Op	Surgical operations
p75NTR	p75 neurotrophin receptor
PLSDA	Partial least squares discriminant analysis
PND	Perioperative neurological deficit
PPAR	Peroxisome proliferator-activated receptor
TPM	Transcripts per million
